# Implantable Smart Technologies (IST): Defining the ‘Sting’ in Data and Device

**DOI:** 10.1007/s10728-015-0309-8

**Published:** 2015-12-08

**Authors:** Gill Haddow, Shawn H. E. Harmon, Leah Gilman

**Affiliations:** 1 Science, Technology and Innovation Studies, The University of Edinburgh, Edinburgh, UK; 2 Edinburgh Law School, The University of Edinburgh, Edinburgh, UK

**Keywords:** Health, Biotechnologies, Medical devices, Smart, Autonomy, Vulnerability, Regulation

## Abstract

In a world surrounded by smart objects from sensors to automated medical devices, the ubiquity of ‘smart’ seems matched only by its lack of clarity. In this article, we use our discussions with expert stakeholders working in areas of implantable medical devices such as cochlear implants, implantable cardiac defibrillators, deep brain stimulators and in vivo biosensors to interrogate the difference facets of smart in ‘implantable smart technologies’, considering also whether regulation needs to respond to the autonomy that such artefacts carry within them. We discover that when smart technology is deconstructed it is a slippery and multi-layered concept. A device’s ability to sense and transmit data and automate medicine can be associated with the ‘sting’ of autonomy being disassociated from human control as well as affecting individual, group, and social environments.

## Introduction

In recent decades, and beginning with the ‘information technology revolution’, multiple so-called technology revolutions have occurred, with the biosciences [6, 10, 23, 30, 45], the nanosciences, and the computing sciences all implicated [24]. The ‘bioscience revolution’ relies on a collection of technologies, techniques and practices that implicate human physiology. Molecular biology (which draws on biochemistry and genomics) and synthetic biology (which designs and constructs artificial biological systems having reference to engineering and computational disciplines), are particularly relevant. While, the ‘revolutionary’ nature of these technological movements can be questioned, their co-evolution and convergence cannot. One of the drivers for this phenomenon is health. As people’s expectations of functionality and ambitions for self-actualisation grow, health grows in significance as a phenomenon of socio-political, economic and scientific concern. In short, mounting and diversifying healthcare pressures serve as critical landscape-shapers for these technology fields [21, 26, 39], making function-restoring and function-replacing technologies increasingly important.

One key ambition is to make diagnostic and therapeutic products that are, if not cheaper (and they are very rarely cheaper), at least smaller, faster, more powerful, less obtrusive, and smart [2, 9, 33, 43]. The first generation of such products comprised devices that were external and removable (wearable or attachable). Examples include glasses and hearing aids for sensory impairments, false teeth, joint braces and prosthetic limbs for functional impairments, and heart-rate monitors, sleep apnea monitors, glucose monitors, seizure and drowsiness onset monitors, and more, for tracking physiological function. In the second generation, devices became penetrable insofar as they exist on the border of the physical body, with components both inside and outside the body. Examples include cochlear implants, insulin pumps, and neuro prosthetics such as electrode array implants. The third and most recent generation of devices are more fully integrated into the body; they are implanted, and where they are not permanently implanted, they are at least very difficult to remove. Indeed, our lives are enacted within an intricate web of increasingly smart technologies, and now these technologies are performing not only *for* us, but also *on* us and *within* us, chipping away at the inaccessibility of our internal bodies. Paradoxically, this internalisation makes the technology both closer to us (in terms of their intimacy with our body), but simultaneously further away from us (in terms of our ability to control what they do to or for us). While a broad understanding of the term ‘implantable’ might include those technologies that are consumed (i.e., pharmaceuticals), our use captures those that are embedded or incorporated into the human body (i.e., an artefact is placed inside the body). Further, while implantable smart technologies (ISTs) may (eventually) include organic implants, our focus is on mechanical devices, which may be more likely to deliver the second aspect implicated by our label: ‘smartness’. Such a reality brings to mind two sage propositions and both inform this paper. The first, from Foucault, offers a view on the fluid, indeed precarious position and nature of ‘man’ (more properly ‘human’), offering an early image of the posthuman:

[M]an is neither the oldest nor the most constant problem that has been posed for human knowledge. … As the archaeology of our thought easily shows, man is an invention of recent date. And one perhaps nearing its end. If … some event of which we can at the moment do no more than sense the possibility—without knowing either what its form will be or what it promises—were to cause them to crumble, as the ground of Classical thought did, at the end of the eighteenth century, then one can certainly wager that man would be erased, like a face drawn in sand at the edge of the sea [11: pp 386–387].

The second, from Baudrillard, is an observation about the apparent human need for obvious otherness. He notes the angst created by ‘invisible robots’, highlighting the perceived danger to human sensibilities of not making their ‘inhumanness’ (or otherness) apparent:

…the substitution in question *has* to be visible: if it is to exert its fascination without creating insecurity, the robot must unequivocally reveal its nature as a mechanical prosthesis (its body is metallic, its gestures are discrete, jerky and unhuman [1: pp 129].

In essence, we are concerned with that slow-developing ‘event’ to which Foucault alludes, an event which might be seen as rooted in the here-and-now and unfolding slowly before our eyes; the decentralisation of the human in contemporary thought. Foucault’s erasure of the human can also be interpreted as referring to a posthuman, or rather to techno-human hybridity resulting in a new mode of being.

In the 1960s, the techno-human was envisaged as having cybernetic mechanisms, made necessary by the demands of future space travel. Authors predicted that space travellers would need a closed-loop feedback mechanism to regulate body responses in a hostile environment, and they offered the term ‘cyborg’ for that entity [7]. Implantable smart technologies included osmotic pumps to deliver drugs and electrical stimulation of both the heart and brain during space travel. No unpleasant after-effects in terms of loss of control is considered as the hypothetical space traveller is unconscious. Thus the increasing reliance and dependence on smart technologies was not seen to threaten an individual’s ability to control the effect (multiple) of internal auto-biotechnologies may have on them. That is why, importantly, we are not necessarily here interested in implantable technologies of the ‘carpentry kind’ such as hip or knee joints, or other static prosthetics, but rather with those unobtrusive technologies that might be considered ‘smart.’ It is these technologies, increasingly powerful and internalised, that have the potential to transform that which is, and give rise to new modes of being (i.e., Foucault’s ‘erase man like a face drawn in sand at the edge of the sea’).

Increasingly intelligent and embedded technologies are playing a more important, and, more importantly, a more ‘normalised role’ in peoples’ lives (i.e., they are on the path to becoming mundane, every day and ubiquitous). The application of such smart technologies has the very real potential to excite the fears cautioned by Baudrillard insofar as they necessitate the implantation of auto techno-devices into the human body, masking and hiding what the device might be monitoring, reporting or interfering with, in, for and on us. What greater ontological insecurity could there be than that created by a device that is autonomous and intimate, and, ironically, through this intimacy, may be out-with individual control and others sight?

Parenthetically, the EU Active Implantable Medical Devices Directive, in Article 2, defines an ‘active medical device’ as any instrument, together with its software, which is to be used for diagnostic and/or therapeutic purposes, and which relies on an electrical or any source of power other than that generated by the body, and it defines active medical implants as any active medical device which is intended to be totally or partially introduced, surgically or medically, into the human body, and which is intended to remain there after the procedure. Exploring the meaning of ‘smart’ within the context of ‘implanted medical devices’, and examining whether the term has any conceptual and analytical currency in relation to specific medical technologies, was an objective of the Implantable Smart Technologies Project (ISTP). The ISTP is an interdisciplinary project that seeks to understand the evolution of implanted health technologies, and to consider some of the more vexing issues thrown up by them. Such as their impact on identity, questions about ownership, control and liability, and whether their development and market authorisation is appropriately regulated. It draws on multiple literature reviews encompassing the technical, social and legal disciplines, and within the project several empirical encounters occurred.^1^ Participants in the ISTP were initially identified through existing networks of researchers, relevant university websites, and local hospitals, and the sample was expanded using a ‘snowball’ approach [40]. Ultimately, eleven interviews were conducted.^2^ We designed a semi-structured interview schedule consisting largely of open questions, which allowed participants to focus on those aspects of the inquiry that most engaged their experience and expertise. The diverse backgrounds of those interviewed offered a rich dataset and suited our flexible approach. All interviews were, with consent, recorded using a digital audio recorder and transcribed verbatim. The transcripts were read multiple times by all members of the team, and analysis proceeded following a broadly grounded theory approach [17], which is arguably now the standard form of qualitative analysis [47]. Under this approach, we individually identified and then collectively affirmed, through an inductive approach, the key themes from the data collected, and then went back to the data for further review and exploration. In the present paper, we have structured our examination of ‘smart’ around the following inquiries [see also 22]:

1. What are the characteristics of an implantable technology that we might want to call smart?
2. Do the ‘smart’ qualities of these technologies have any social or practice implications?

First, drawing on our empirical data, we unpack the idea of ‘smart’ in relation to four specific implanted devices, namely cochlear implants (CI), implantable cardiac defibrillators (ICD), in vivo biosensors (IVBS), and deep brain stimulators (DBS), each of which were singled out during data collection as exemplars of current ‘smart’ technologies. We conclude that the term holds multiple and not always compatible meanings. Second, we suggest that ‘smart’ can carry a ‘sting’, or rather multiple stings relating to, on the one hand, being complex and responsive, and on the other hand, igniting concerns about lack of control and vulnerability. We end with the thought that *smart technology can give autonomy whilst simultaneously taking it away*. However, the (partial) erasure of the (techno)-human is a highly variable process partly related to the functionality and accessibility of the implantable smart device.

## Defining ‘Smart’ in ISTs

### The Data

We began our interactions with participants, by asking whether there was an awareness of the term ‘smart’ in relation to devices. Responses were mixed, with a few admitting that it was not a term they used in everyday discussions about devices, but with most admitting that, even if they did not use the term in relation to ‘implantable smart technology’, they had heard it and could articulate their conception of ‘smart’. As Respondent 2, an engineer, suggested:

Smart technology is where you’re using information, I would say smart technology is where you get information from sensors, or from imaging, or from some other way of measuring what’s going on, and you make a decision based on that.

In vivo biosensors (IVBS) is an example of such a technology ‘getting information’, which is currently in development to enhance radiotherapy treatment for cancerous tumours. Many tumours can be effectively treated with radiotherapy however some are stubbornly radio resistant. Biosensors may be able to demonstrate resistance by undertaking biological measurements of the tumour environment assessing whether real time fluctuations in oxygen, pH levels, etc., could be exploited to optimise the timing of treatment to overcome radiotherapy resistance. That is the patient’s radiotherapy treatment can be scheduled for when the tumour is at its least resistant [51]. The idea of complex information gathering was then added to and built upon by other respondents, with a ‘doing something’ function. Respondent 9, a regulator, stated:

In a regulatory context, I’ve never heard [smart] used. … What would it mean to me? I suppose … it would be something along the lines of … these type devices, but ones that are more sophisticated. I would say that, if they’re implanted and they’re smart, then they’ll act on chemical signals within the body that would then end up doing something else. … A lot of these do similar sorts of things: pick-up signals within the body and then do something. … You’d assume that ‘smart’ is looking at probably a wider variety of factors and maybe could do more than one thing as well.

Similarly, Respondent 8, a bioethicist stated:

I think this term smart just depends on how people want to use it actually. It seems to me it’s got to do with complexity, it’s got to do with *responsiveness*.

Within the quotations above, we begin to see some evidence about what smart might mean in real terms; a mention of complex information gathering and the need for ‘responsiveness’. To these can be added ‘autonomy’, and of ‘by itself’ as suggested by Respondent 10, a government researcher, who stated the following:

What would I envisage smart being? … Smart gives you the impression that you’re implanting something which is going to do something itself which the body might not be able to do. That’s how I suppose I would envisage what it means … Because it’s something that’s being implanted in the body in order to help it do something it’s not [doing], I suppose. But I see that, and from the purpose of smart, I see that as that sounds smart to me, more advanced.

Given the above, it is important to explore and unpack the key ideas of ‘complexity, ‘responsiveness’ and ‘autonomy’. ICDs are small devices which are surgically implanted in the chest and which use electrical pulses and shocks to help control life-threatening arrhythmias, especially those that can cause sudden cardiac arrest [3]. There are similarities between ICDs and IVBS, in that the patient has a foreign mechanical object inserted into the body. They differ, however, in that whilst the latter may not be noticed by the individual, ICDs provide painful shocks at unpredictable times. ICDs and pacemakers collect data about the functioning of their immediate environment (e.g., the heart), and provide therapy (e.g., releasing electrical pulses and/or an electric shock) in a responsive manner, and so are viewed as more ‘active’ than valve replacements. Our medical respondents felt that (ICD)s in particular were smart in part because they were “intelligent”. Respondent 6, a physician, stated:

Pacemakers and implantable defibrillators [are] checking what the body’s doing, and if it’s not doing it, they will take over, so it has almost got an artificial intelligence element to it, and it’s got decision-making algorithms that if this happens then you will do this, if this happens, it will do that.

On this basis, they are more active and have a degree of complexity, responsiveness and autonomy not found in say, a heart valve replacement. As mentioned such attributes were necessarily related to a ‘closed loop’ system originally envisaged in the utopia of future space travel. Respondent 2, stated of glaucoma sensors that were previously in development:

It would be a closed loop. The way that would work, the chip would be in there, so inside there you would measure pressure then almost certainly transmit that out [to] a computer. But the computer would then have to decide, ‘Is this abnormal for this person? If it is, we need to release some glaucoma drug. So then it would transmit back into say, ‘Release the drug’. So it is a closed loop.

Importantly, the loop need not be contained solely within the body. On this, Respondent 5, a lawyer stated:

It seems like an irrelevant contingency if it was wirelessly sending something to be processed on a laptop on the other side of someone’s hospital room or something, and then wirelessly getting information to perform in some way. That seems just as smart as if the processing was being done within a bigger, more complicated, more heavily powered device, all inside the person. That doesn’t seem to be an interesting distinction that takes away its smartness, just because there was wireless communication with some more processing power somewhere else. It does seem to be less smart if all [the device] is doing is giving information to a clinician who then goes and gets a syringe and delivers treatment. That seems to be just monitoring, [which] isn’t smartness. It’s about the responsiveness but some of that responding loop could be on the other side of the room, or the other side of the world.

So the functions performed by the device could be performed both internally and externally, but the point is that the ‘loop’ is closed to the patient and physician; decisions are made by the device or the system that the device is ‘plugged into’, which also highlights (1) the ‘autonomy’ element to the device and (2) the loss of human control.

Yet, for the person who is using the smart device to enhance quantity or quality of life, there are different consequences for individual autonomy dependent on the functioning and capability of the device itself. Hence, returning to Foucault’s ‘erasure’ of the (hu)man, we can argue that partial erasure depends on the effects of loss of physiological control. This is, to a certain extent, is dependent on the intended functioning of the device.

On autonomy, and loss of physiological control, IVBSs and cochlear implants (CIs) exhibit considerably different levels of smart, say, to that of ICDs, for example. CIs, which are penetrative but not truly implantable, can provide a sense of sound to those who are profoundly deaf or extremely hard-of-hearing. An external component comprising a microphone, a speech processor, and a transmitter, sits behind the ear, and an internal component comprising an electrode array is surgically placed within the ear to stimulate the auditory nerve [14, 42]. CIs do not restore ‘normal hearing’, but rather replace it by interacting with the environment and the auditory nerve. For Respondent 1, an analogy was drawn between the device and a doctor:

So, something that somehow expresses that continuousness between a little tiny very skilled but highly specialised doctor inside you talking to a more skilled doctor outside you who then consults a whole team of [specialists] to say what to do next.

Connected to both artificial intelligence and autonomy was the idea that the device makes a contribution to the clinical care of the patient; it must serve a useful healthcare function. Respondent 3 stated:

I think it probably makes [the device] smarter if it leads to an improvement in treatment. I think you can’t really just use these devices for, ‘Isn’t it clever we’re sensing something, but we can’t do anything about it’…so I think it has to probably be a closed loop to improve the clinical result.

This idea that the device would serve some active therapeutic function was shared by most respondents. In other words, to be smart, the device represents something more than the previously mentioned ‘carpentry of implanted replacements’; it can be more than the artificial limbs, joints, or valves that replace existing mechanical elements of the body. Rather, it makes treatment more quick and effective. For some, such as Respondent 7, a medical professional, this idea of making treatment quicker and more effective drew ICDs, and, interestingly, CIs into the ‘smarter’ category beyond that of IVBS. On the contrary, for others, such as Respondent 2, this active use of information in a responsive manner is what differentiates ICDs, for example, from CIs or IVBSs; for unlike the other technologies, ICDs provide a therapeutic intervention autonomously based on information it obtains within the body. Respondent 3, concurred, stating:

A lot of these are put in for specific correction of function. Pacemakers, cochlear implants, they’re not really *sensing* as such. The abnormality is already known, and it’s there to correct it, or kick-in if it’s detected. Whereas, some of these newer smarter [devices], I think, are [in] more fluid situations. There’s more uncertainty around the readings and what they’re going to mean, and when they’re going to need to be used. I can imagine the glucose sensor and insulin pump would give varying amounts of insulin day to day because there’s going to be varying activity, for instance, going on in the patient, so it’s a much more dynamic, fluid situation [emphasis added].

Without this aspect of automation of the therapeutic component, some questioned the basis for considering a device ‘smart’. In this regard, truly smart devices such as ICDs could be differentiated from CIs and IVBSs.

Thus, for a smart response to be ‘smart’ it needs to be more than just on/off; it usually needs to operate at multiple or varied levels. Increased smartness can be about increased automation (quicker responses which minimise role of clinician/patient), and it can be linked to the complexity of what is being sensed and responded to (e.g., measuring multiple variables (which may be technologically difficult to measure), processing and delivering an appropriate response. Individuals, like Respondent 3 who emphasised importance of closed loop, seem to emphasise the first as a main condition of smartness (autonomy/automation), and others, like Respondents 1 and 2, seem to emphasise the second condition (complexity of responsiveness). In the case of CIs, ‘human’ autonomy is not threatened in a way that it might be by an ICD. The person can remove the outside monitor from their ear for example. Further, while there was a powered transmitter that sent radio frequencies to the brain for processing, the ‘smart’ element came from the human brain:

… The brain adapts to that way of hearing … through a process of us fine-tuning the devices, because it’s not just one appointment, they come to see us about seven times in the first year just for us to fine-tune it and to keep the levels, like up here and they’re backwards and forwards.

Hence, Respondent 4, a CI specialist, confirmed that success with CIs was “probably 15 % technology and 85 % person; the things that are important are good surgery to put the implant in place, good programming to tailor the device for the individual, and then individual patient compliance, perseverance and luck.” Baudrillard’s analysis of the ontological insecurity of an implanted modifications is a key starting point to examining the effects of invisible technologies. Although where the recipient of the information from the closed loop system may not matter, as discussed earlier, the fact that the device is inside the body and therefore, ironically, out of reach contributes to the lack of control. CIs are still partly controlled by the recipient removing the transmitter that is placed behind the ear; a transmitter that when it is worn is visible. Baudrillard’s insight is that the invisibility of the smart device causes angst in the bystander. And although Baudrillard demonstrates the darker side of implanting biotechnologies, he does not discuss this in relation to recipient consequences and the varying effects that different invisible technologies can have on those they are implanted in. Closed loop systems that are inserted into the interior of the body offer therapy through the ability of the device to sense and respond autonomously. But there is a ‘sting’ that comes from the smart, and we turn to that shortly.

### The Implications

So what can we surmise more broadly from the above evidence? At base, given these responses, smart is a complex concept that holds multiple meanings. For some respondents, the term smart was not viewed as especially helpful. Respondent 2, an engineer, said that ‘smart’, and:

… words like ‘micro’ and ‘nano’, get used and abused widely. It’s not a terribly helpful word because there are some things that are very smart and require a great deal of machine intelligence and processing.

For others, depending on the criteria focused on (as between intelligence, autonomy, and responsiveness), one might categorise devices differently (i.e., as ‘smart’ under one criteria but as ‘not smart’ under another; a CI is not smart as it does not have a sensory ability, but it is smart as it delivers a therapy; a DBS and ICDs are smart as they are both sensitive and responsive). All this makes authoritatively categorising devices both difficult and problematic. Also when respondents were discussing *particular* technologies as opposed to “smart” in the abstract, they were not necessarily consistent about what were the most important facets of smart. Their assessment of smartness *in practice* was contingent on other factor, such as the newness of the technology (hence ICDs were seen as smarter than pacemakers), familiarity with the technology (some participants were normalising what they were working on and with and therefore tending to have views on auto biotechnologies they knew less about as more complex. For example Respondent 2’s familiarity with IVBs but not DBS.) Relatedly, where it was placed in the body could influence whether something was also viewed as more or less smart, though this wasn’t usually mentioned in the abstract discussions of smart (e.g., DBS tended to be seen as smarter as it was located in the brain and was inaccessible whereas CIs were sometimes viewed as less smart because their location was not thought significant and they were accessible.

**Fig. 1:**
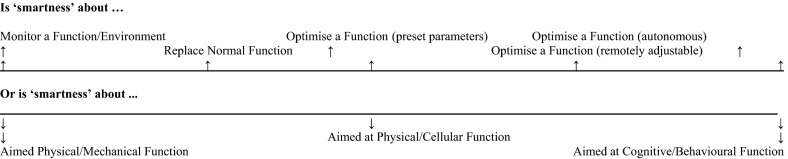
‘Smartness’ continuum

Despite, or rather because of this, we settle on a definition of ‘smart’, that makes more sense to think of smart along the lines of a continuum (see Fig. 1), with those devices that sense on one end and those that activate therapeutic responses that are inaccessible and out-with the control of the individual and the immediate oversight of health professionals on the other. Binding the continuum together is the idea that smartness necessitates some degree of autonomy within the device (i.e., some independence from human participation either by recipient or other in a set function) coupled with one or more of the following actions:

- sensitivity (i.e., record and transmit information about an environment. Increased smartness is linked to complexity of what is being sensed and processed);
- responsiveness (i.e., reacts to changes in the environment and amends functions hence increased sensitivity linked to level of automation/closed loop);
- autonomy (i.e., performing functions the body wouldn’t normally perform).

At one end of the continuum, smartness demands replacement of mechanical or sensory functions, generally allied to either preparation of data for internal processing, or transmission of data for external reporting and analysis. A higher level of smartness would include the possibility of direct or remote updating and adjusting of the instructional code which underlies the device (i.e., the software), thereby allowing the device’s actions to be modifiable (or the scope of the monitored criteria to be alterable). The highest level of smartness would involve the device being able to alter its monitoring criteria based on an autonomous assessment of its environment, and to adjust delivery of treatment based thereon. At the other end of continuum introduces a qualitative change in the way that devices implanted in the patient relate to the body; they manage, and will soon deliver, a sensory-based automated treatment platform that reacts to changes in their physiological environment.

## The ‘Stings’ in ‘Smart’

Given the expectations forming around these smart biotechnologies, smart might come to demand that the device be able to:

- sense complex and dynamic physiological settings subject to multiple changes;
- measure these potentially subtle changes and collate the data;
- transmit this dynamic information in real-time and;
- react to the changes in real-time by providing alterations in therapy (also delivered by the device).

Such devices shift treatment well into the sphere of ‘automated medicine’, but key technical hurdles remain in relation to their practical application in the clinical setting, not least being the need to harness sufficient power for sufficiently long periods of time, and to store (or have ready access to) sufficient treatment compounds to give them appropriate endurance. The issue of powering such complex devices is obviously a technical ‘sting’ that is being worked on (and was highlighted in the first empirical encounter we held: the workshop in 2009). However, there are also some other important ‘stings’ associated with ISTs that might be classified as social and regulatory. The two most obvious ones are (1) decreased intentionality, and (2) increased vulnerability, to which we now turn.

### Decreased Intentionality

First and foremost, as foreshadowed by the above references to closed loops (which, of course, have some positive implications), there is the real possibility that the physician could recede from the treatment relationship and simultaneously lose control over the medical intervention (recognising, of course, that that control is typically exercised in collaboration with the patient). While the physician would (presumably) be implicated in the initial programming of the device (i.e., articulating the parameters of ‘normal’ functioning), much of medical monitoring and action afterwards would be left to the machine intelligence. In this way, the physician is less ‘in touch’ with the treatment regime and the patient, and so is potentially less sensitive to the patient’s needs, including the need for the patient’s device to be adjustment. This reduction in the physician’s intentionality and immediacy could lead to an erosion in the doctor-patient relationship, which often relies on points of contact and positive interaction in order to build the trust needed for the most constructive relationships. At base, this means that, if the concept of ‘compassionate care’ is to drive healthcare forward (and that is certainly the policy objectives at the moment), then serious thought will have to be given with respect to how these devices enter into and reshape the clinical relationship.

Additionally, there may be a loss of control on the part of the patient, and this can diminish the responsibility the patient feels toward their own condition. Frequently presented as a ‘pacemaker for the brain’, deep brain stimulators (DBSs) comprise electrodes implanted in the brain, a pulse generator implanted in the chest (near the collarbone), and a subcutaneous wire connecting them. Intended to alleviate tremors, stiffness, and slowness caused by Parkinson’s disease, reports suggest that DBS may have implications for improving lung function, memory, and mood disorders such as depression [34]. DBSs have been the subject of intense investigation; studies have uncovered (1) very different expectations for, and tolerances about, chronic illnesses and the side effects of their treatment [16, 49]. (2) The variety and progression of emotional response to DBS, and (3) the need for greater cooperation between stakeholders to create realistic public perceptions of DBS [15, 37]. DBSs have also been the subject of legal concern, for they have been known to cause significant personality change, which can have implications for capacity [28]. So, for example, with monitoring and treatment removed from the patient and located in the implanted device, the patient might take less seriously the personal responsibility of adopting steps to otherwise maintain a healthy condition. On this, it has been argued that, although DBSs can significantly improve symptoms, they also take control out of the hands of the patient/person who has been managing their illness up to then [16].

Ultimately, the reduced need, and indeed the reduced ability (because the device is inside you and so ‘out of reach’) to monitor conditions, or to actively/intentionally take medication or take conscious steps to follow instructions, the patient may feel less of a connection with the treatment regime, and this might have knock-on effects for the ultimate effectiveness of the intervention. Significantly, CIs have been the target of heated debates and the subject of much research, most likely as a result of the following factors [4, 5]:

- they have been in use longer than the other subject ISTs and so are more pervasive [31];
- they have been designed for a deaf community that has a strong cultural identity that is linked to their physiological condition [44, 48];
- they have been controversially implanted in children as young as 6 months old [8, 36].

In any event, CIs have profound life and socialisation implications, which has resulted in strong resistance in some circles and hence has produced an affirmation, in some cases, of individual and group deaf identity. In other words, the patient might experience the same powerlessness (or functionless-ness) that the physician might feel, thereby further contributing to a weakening of the healthcare partnership that supports the best health outcomes. Again, this is not a given, but the potentiality of this requires us to pay attention to how devices impact on patient behaviour and clinical relationships as they become more ubiquitous.

### Increased Vulnerability

In CIs, stress after implantation has been reported due to expectations of the device being unmet, the difficulty in tuning the devices and continuing communication/discipline problems associated with childhood deafness. CIs are often given on the assumption that congenital hearing loss is cochlear in origin; some misassumptions have been made with the result that children have been given implants that will not work. The device can lead to ‘non-auditory stimulation’—where nerves around the site are stimulated by the electrical energy, and this can lead to facial twitching. Tied to the phenomenon of decreased intentionality, we might add that of increased vulnerability. With respect to patients, the loss of control can lead to feelings of vulnerability that are not dislodged by the trust they might nonetheless feel toward the device [and the physician(s) who prescribed and implanted it]. Respondent 3 a medic, stated “Yes, you’re handing over responsibility to a device…but over time I guess you probably wouldn’t, you’d just trust it.” But Respondent 6 stated in relation to ICDs:

[D]efibrillators [are] a mixed blessing really. On the one hand, it’s reassuring [that] it’s there, and if you need it, it goes off, but if it does go off, either it’s gone off correctly—but that brings its own concerns of, ‘Why did it go off?’ ‘Am I about to die?’ ‘Is something terrible going to happen to me?’—or it goes off inappropriately, and it’s quite painful, and you think, ‘*I haven’t got any control over it, I can’t stop it’.* And that can be quite difficult for patients.

The firing of an ICD imposes a ‘dual shock’ in many cases; there is the emotional or psychological shock of its sudden operation and the comprehension that something has gone wrong within you, combined with the physical shock (i.e., the sudden painful sensation) of its function. Respondent 11, a device recipient, described this as follows:

The suddenness. Always just the suddenness of it. There’s no warning. I’m saying no warning, maybe just a minute or two [before] you say to yourself, ‘I’m not right’, and then it kicks in. You know in yourself, ‘I’m not right’; I know anyway, something’s going to happen. … I can’t even say it’s a physical thing.

In essence, as the case with both ICDs and DBS show, there could emerge a sense of being at the mercy of not only the foibles of one’s (failing/ailing) body, but also the programmed actions of the device. In short, the device has an unpleasant intervention but also signals an untoward event, knowledge of which can be disconcerting. Implantation of ICDs is major operation and there are lifestyle and physiological adjustments to be made. Patients are dependent on the device to rescue them in the event of sudden heart arrhythmias. ICDs are known to cause anxiety and depression in patients [25, 27, 35, 41, 46], avoidance of physical activity and sexual contact, and effect family relationships. The more shocks felt by the patient the higher the anxiety [50, 52]. As well as the autonomy of the technology linking to the patient’s lack of control, Baudrillard’s point made earlier about visibility could again be highlighted here. In the case of ICDs, the technology is almost invisible (only making its presence felt on the occasions it is activated) leading to the unsettling lack of clarity about the human/technology distinction.

As mentioned, IVBS are less intrusive than ICDs and their functionality was viewed by some of our participants as less ‘smart’ due to the devices lack of a closed loop system. Indeed, early social science research with recovering prostate cancer patients demonstrates a willingness to accept IVBSs, and even an enthusiasm for a more ambitious functionality that goes beyond a beacon system (i.e., identifying the timing and location for radiotherapy) and toward an IVBS that is a long-term surveillance system to assess the reappearance of cancer tumours. Having said that, periods of ‘acclimatisation’ were mentioned as being necessary [18], and willingness to have an IVBS may vary depending on the circumstances around their implantation and the concomitant evaluation of what will be lost and gained from the IVBS? That is, some loss of autonomous control and increased vulnerability versus sensing and responding to a tumour recurrence.

A second aspect of vulnerability is that relating to the security of the device, which has obvious health implications for patients, but also liability implications for healthcare administrators and device designers. Moreover, as the devices become smarter, gather more data, and become more interconnected, there arise more concerns about data security (and system hacking). Not all our respondents were equally concerned about this issue, particularly for devices that were merely monitoring internal processes. Respondent 2, an engineer and technology developer, stated:

The data becomes insecure, as soon as it leaves this device … but that’s not an implant problem, that’s a general medical data problem. The inside-the-body stuff I don’t think is a concern. The concern kicks in when you get outside the body and you start doing something with the data. Because nobody can get at the data unless they’re right up against you. I suppose, potentially, if someone really wanted to measure your tumour hypoxia while you’re on the Underground they could press something up against you and measure it. We’re into the silly world there.

Despite this cynicism around the motivation and value of hacking, other respondents raised this issue of patient privacy and security. Respondent 1 stated:

As a lawyer, what I would be interested in is the extent to which [the device] could be, or is, controlled remotely, for example. Or its ability to self-regulate, and in what ways, and if it is self-regulating, then what other controls could be exercised over it if it became inappropriate or unsafe.

A team in America demonstrated that a wireless reprogrammable ICD or pacemaker could be hacked, the information it contains changed, and its settings altered, all using readily available radio devices and software. The SWAMI report outline scenarios where “remote homicide” may be possible by hacking into such devices and disrupting their software or signals to give wrongful treatments, or prevent emergency signals being responded to [12]. Respondent 7, a medic, stated that, although pacemakers can be interrogated online, they currently download their information to a server but do not typically upload instructions, largely because of security concerns:

The pacemaker companies, I think, are worried that if their pacemaker is compromised then they could be liable, and it might be perceived that they haven’t made their device secure enough for the patient. So at the moment, all we can do is obtain diagnostic information, but we can’t reprogramme pacemakers remotely.

Despite different levels of concern in our sample, matters of privacy and security are not fanciful. Tens of millions of patients worldwide rely on implanted medical devices for life-critical functions, and our respondents acknowledged that the number of IST-reliant patients will rise because “every physiological function is a system, and if you can monitor every system you will be able to improve healthcare” (Respondent 3, medic). However, the appreciation of device security and its appropriate balance with utility, which encompasses issues such as:

- device identification by authorised entities;
- data access and device reconfiguration by appropriate entities;
- data accuracy;
- software upgrading by appropriate authorities;
- multi-device coordination and communication; and
- manufacture audit capabilities in the event of failure,

is only in its infancy [20]. The possibility of hacking into devices has already been demonstrated [19, 38], and the list of vulnerabilities derivative of increased IST connectivity is growing [29]. Responses to these issues must be both technology and regulation based, but to date standards have not been well specified [13, 32].

## Conclusions

In an environment surrounded by technologies defined as smart, it is important to refine what it is that offers the most benefits and also risks from such interventions inside the human body. We have discussed how smart in the context of medical technologies such as ICDs, IVBs, CIs and DBSs can be thought of as relating to autonomy, responsiveness and complexity. However, the ability to manage a physiological feedback mechanism—the so called ‘closed loop’—originally featuring in how humans could cope with space travel and the genesis behind the term ‘cyborg’—featured highly in respondent accounts. Yet regardless of the smartness of the technology, each of these implants affected individual, family, and group relationships in unintended and diverse ways, whether it was anxiety and depression in some ICD patients, identity challenges for DBS, resentment in areas of the CI group, or needing to ‘acclimatise’ in some potentially unique ways to an organic/inorganic status, cyborg or techno-human in the IVBs patients.

One possible interpretation of the word smart not mentioned in accounts, is when smart is used as an adverb to mean a sharp, stinging pain. Hence, the ‘stings’ associated with smart implantable technologies are that the devices themselves are out-with the control of the implanted individual and often his or her physician, thereby reducing human intentionality, and with increasing complexity and connectivity, generating new vulnerabilities for the patient and healthcare providers. However, we have come to believe that taking experiential views and variable individual and group reactions to implantable smart technologies enriches philosophical discussions. Both Foucault and Baudrillard, for example, point to the ontological status of the human; the former to the possible erasure, and the latter for the angst caused by a process of indiscernible technological replacements. In a sense, we point to the unevenness of such a trajectory, and to the partiality of application as well as ambivalent compliance. We feel this is sometimes missed in theoretical discussions of the post human, and this was the case in the earlier discussions, and we have interpreted from Foucault but may possibly apply to other later work. We accomplished this philosophical enrichment by using data from interviews with stakeholders to address the question of what is smart in ISTs. It was never our intention to produce a statistically generalizable result that would represent an evidence base on which other technologies can be assessed or judged. On the contrary, this exercise demonstrated that it is possible to investigate the current landscape by identifying those involved and producing an account of social, political and legal issues that is, in this case, based on the ‘inside views’ of those involved.

This leads us to argue that implanting medical technologies cannot be separated from the social practices and broader environment in which they take place. Much has been written about ‘technological affordances’ and the middle ground between an under-socialised or over-socialised view of technology seeking to locate the consequences of the technology within its current social setting—smart devices offer opportunities to enhance both the quality and quantity of life yet at the costs of control and heightened vulnerability. Further, implanted technologies are physically hidden but their affordances—their actions—emerge during the interactions with human practice. ‘Action possibilities’ are therefore simultaneously consequentialist being both positive and negative in the same way that smart technologies are autonomous but also stinging and smart. We conclude that ‘smart’ relies on the presence of several attributes (e.g., autonomy, responsiveness, complexity) and is described by a continuum. Understanding the elements of smart will allow for the better regulatory classification of devices and the issues to which they might give rise.

## Footnotes

1 An initial pilot workshop in 2009; semi-structured qualitative interviews with thought-leaders and stakeholders in the field in 2013–14; and close collaboration with an artist (from 2012 to 2014) who both helped shape the interview process and generated interpretive artistic outputs relevant to our findings. For more on the ISTP, see http://masoninstitute.org/our-research/implantable-smart-technologies-technical-social-and-regulatory/.

2 Five with professionals working at the legal/ethical interface of medical technologies, three with medical practitioners and researchers, one with a clinical scientist, one with an engineer, and one with a patient reliant on an implanted device.
